# Maintenance of pluripotency in the entire ectoderm enables neural crest formation

**DOI:** 10.21203/rs.3.rs-2285117/v1

**Published:** 2023-01-25

**Authors:** Ceren Pajanoja, Jenny Hsin, Bradley Olinger, Andrew Schiffmacher, Shaun Abrams, Arvydas Dapkunas, Zarin Zainul, Andrew D. Doyle, Daniel Martin, Laura Kerosuo

**Affiliations:** 1 National Institute of Dental and Craniofacial Research, Intramural Research Program, Neural Crest Development and Disease Unit, National Institutes of Health, Bethesda, USA; 2 University of Helsinki, Faculty of Medicine, Helsinki, Finland; 3 National Institute of Dental and Craniofacial Research, Intramural Research Program, NIDCR Imaging Core, National Institutes of Health, Bethesda, USA; 4 National Institute of Dental and Craniofacial Research, Intramural Research Program, Genomics and Computational Biology Core, National Institutes of Health, Bethesda, USA

**Keywords:** neural crest pluripotency, multiplex spatial transcriptomics, single cell analysis, ectodermal patterning, broad ectodermal pluripotency, ectodermal domains, pan-ectodermal stem-cells, post-gastrulation pluripotency, neural crest stemness

## Abstract

The ability of the pluripotent epiblast to contribute progeny to all three germ layers is thought to be lost after gastrulation. The later-forming neural crest (NC) rises from ectoderm and it remains poorly understood how its exceptionally high stem-cell potential to generate mesodermal- and endodermal-like cells is obtained. We monitored transcriptional changes from gastrulation to neurulation using single-cell-Multiplex-Spatial-Transcriptomics (scMST) complemented with RNA-sequencing. Unexpectedly, we find maintenance of undecided Nanog/Oct4-PouV/Klf4-positive pluripotent-like pan-ectodermal stem-cells spanning the entire ectoderm late in the neurulation process with ectodermal patterning completed only at the end of neurulation when pluripotency becomes restricted to NC, challenging our understanding of gastrulation. Furthermore, broad ectodermal pluripotency is found at all axial levels unrelated to the NC lineage the cells later commit to, suggesting a general role in stemness enhancement and proposing a mechanism by which the NC acquires its ability to form derivatives beyond “ectodermal-capacity” in chick and mouse embryos.

## Introduction

**Figure 1 |:**
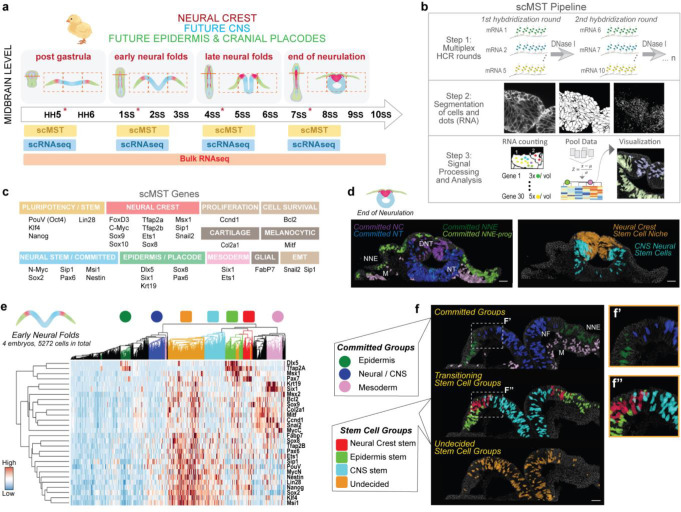
Multiplex Single Cell Spatial Transcriptomics reveals pluripotent-like cells broadly throughout the neurulating ectoderm. **a**, Experimental plan and a schematic of neurulation demonstrating the rise of neural folds and how ectoderm is patterned into three domains. Dashed squares demonstrate fields of views for scMST. All twelve stages were used for bulkRNAseq and asterisks highlight the four stages that were used for scMST and scRNAseq. **b**, scMST pipeline demonstrates serial hybridizations rounds, segmentation of cells and individual RNA transcripts (dots). Then, pooled data of transcript counts in individual cells is pooled into a heatmap followed by back-mapping of cells in the subclusters to visualize their location in the original tissue sections by pseudo-coloring the cells (see methods for details). **c**, List of genes chosen for scMST. **d**, Pseudo-colored scMST subpopulations at the end of neurulation. **e**, scMST results from early neural fold stage highlight broad existence of pluripotent-like cells throughout the developing ectoderm. Heatmap shows cells clustered into transcriptionally defined subpopulations labeled with a color. Circle symbols indicate committed cell groups and square symbols indicate stem cell groups. **F**, Each single cell is mapped back into original position in the embryo image and pseudo-colored according to their representative subpopulation as labeled in the heatmap. **f’**, Higher magnification of committed subpopulations revels that the cells of different groups do not overlap spatially, whereas **f”**, transitioning stem cell groups show spatial overlap. NNE=non neural ectoderm, DNT=dorsal neural tube, NT=neural tube, NF= neural fold, M=mesoderm. Scale bar, 30 μm.

Pluripotent epiblast stem cells, with the potential to become every cell type in the body, become restricted to germ layer-specific fates during gastrulation. Morphogen gradients polarize the body axes leading to proper tissue patterning. Medial-lateral patterning of the ectoderm germ layer results in formation of the medial future central nervous system (CNS) domain flanked by the lateral non-neural ectoderm (NNE) domains that become the skin (Fig. 1a). The neural plate border (NPB) between these two domains gives rise to the neural crest (NC) and cranial placodes. The NC represents an exception to the idea of germ layer restriction because in addition to giving rise to ectodermal-like derivatives such as the peripheral nervous system and pigment cells, it also differentiates to facial bones and cartilage, smooth muscle, adipocytes, and endocrine cells – cell types that typically arise from the mesodermal and endodermal germ layers ^1^. How the NC establishes this exceptionally high pluripotent-like stem cell state remains poorly understood.

Two contradicting hypotheses have been recently proposed to explain this intriguing conundrum. One model suggests the exceptionally high stemness of the NC is retained from blastula stage (the Xenopus animal cap ectoderm, equivalent of the mammalian epiblast) mainly based on continuos expression of key NC genes that promote pluripotency^2^. A more recent study used Wnt1Cre and Oct4 reporter mouse lines to conclude that rather than maintaining pluripotency, the high level of NC stemness is enabled by the unique ability of the NC to re-activate the pluripotency program during the neurulation process at late neural fold stages well after gastrulation is completed^3^, a hypothesis also supported by pseudo-time analysis in a scRNA sequencing study of whole frog embryos^4^, and ectopic expression experiments of *Ventx2* and *Oct25* (frog homologues of *Nanog* and *Oct4*) in the ectoderm outside the NC domain^5^. We recently used the previous version of the method we developed, single cell Multiplex Spatial Transcriptomics, scMST (Fig. 1b), to investigate NC stemness at the end of neurulation in the chick dorsal neural tube^6^. The results revealed a subset of NC cells alongside the dorsal midline that co-express *Nanog*, *PouV (Pou5f3)/Oct4* and *Klf4* transcription factors^7^, which are core components of a gene module that drives pluripotency in embryonic stem cells^8–10^. We defined this domain as a transient NC stem cell niche^7^. Recent studies have since confirmed the expression of pluripotency genes in the NC also in mouse and frog embryos^3,5^. However, defining the time at which the NC acquire stemness is critical for determining whether it is maintained or reactivated. Understanding how NC forms is highly clinically relevant : ~30% of all birth defects comprise of neurocristopathies, and the demand for using NC cells for regerative purposes is increasing. Furthermore, understanding of whether climbing upward the Waddigton’s epigenetic landscape^11^ truly is part of embryogenis will provide important clarity to the comprehension of pluripotency regulation in normal as well as malignant tissue growth.

To this end, we investigated the temporal changes in stemness genes to gain insights into how the developing ectoderm can acquire a domain with high, pluripotency-like features so late in development. Multiple high-resolution approaches, including scMST, single cell, and bulk RNA sequencing (scRNAseq, bulk RNAseq) were combined to monitor gene expression across a broad series of developmental stages for a comprehensive analysis at the midbrain level (Figure 1a), as this region of the NC gives rise to a diverse set of cell types including the craniofacial skeleton.

### Spatial transcriptomics reveals involvement of pluripotent-like stem cells in ectodermal patterning

30 genes that reflect all three ectodermal domains (NNE, NC, CNS) as well as pluripotency factors, were chosen for our analysis (Figure 1c). Due to the small contribution of the midbrain region to placodal development, placodes were not addressed in the analysis. To analyze the entire ectoderm, the computational pipeline for scMST gene expression analysis was optimized for both multiple developmental stages and several fields of views captured per section (Figure 1a). Individual cells of each developmental stage (collected from four embryos per stage and consisting of 4866–8253 cells per stage) were pooled into a heatmap and hierarchically clustered into transcriptionally distinct subpopulations. Based on their transcriptional profile, each subpopulation was annotated and assigned a color. Then each cell was visualized by mapping it back to the original embryo image by pseudo-coloring the cells on the sections with the same color as their respective subpopulation in the heatmap, herein referred to as spatial back-mapping (Figure 1, b–f’ Supplemental materials and methods, Figure S1a- f’ and 2). We analyzed stemness gene expression at four different stages from gastrulation to the end of neurulation, starting with the oldest stage. Cells in the dorsal neural tube (DNT, 7 somites) at the end of neurulation, contained two stem cell populations (Figure 1d and Figure S1c-c’): 1) NC stem cells (orange), which co-expressed NC markers but also genes of the two other ectodermal domains alongside pluripotency genes *Nanog, PouV/Oct4, Klf4* together with *Lin28,* and 2) neural stem cells (light blue) that have a predominantly CNS profile, but also co-expressed pluripotency genes and a low level of NC genes. We speculate that this unique gene expression profile may provide plasticity to the DNT as it goes through major reconstruction to form the roof plate after the NC has emigrated from the neural epithelium^12^. The NC stem cells also co-express neural, glial, pigment and mesenchymal genes suggesting that at this premigratory stage they remain unspecified regarding their future NC lineage. Importantly, the DNT also consists of committed cells, including NC cells that do not have a stem cell profile and only express NC genes (Figure 1d and Figure S1c-c’), in line with our previous findings^7^.

To understand how NC stemness arises between gastrulation and neurulation, we analyzed the ectoderm at three different developmental stages that encompass neural fold elevation (Figure 1a, Figure S1b). At late neural fold stage (4 somites), we detected committed cells (circle symbols) of all three domains in the correct spatial positions, which notably, do not show spatial overlap (Figure S1d-d’). Unexpectedly, at this relatively late post-gastrulation stage when the ectodermal domains are thought to be spatially restricted and fully committed, we discovered stem cell subgroups that co-expressed the pluripotency genes (square symbols). Based on their gene expression profile, we detected transitioning stem cells in all three domains: both CNS and the NNE stem cells still also expressed some NPB markers, whereas NPB stem cells expressed a broad array of genes including markers of both neighboring domains. Importantly, the cells in these subpopulations also spatially overlapped with each other in the ectoderm reflecting a transitioning status (Figure S1d’). Finally, we identified an additional stem cell group with an uncommitted transcriptional profile that spatially was mostly located in the NC and CNS domains (orange cluster in Figure S1d’). Combined, these results suggest that cells in each subdomain of the ectoderm at late neural fold stage have much higher plasticity than previously known.

Next, we analysed embryos at the early neural fold stage (1 somite). Intriguingly, we detected evidence for even broader stemness across the entire ectoderm: while some committed cells were found at their anticipated locations in the future epidermis and CNS, the majority of the cells met the criteria of a stem cell as evaluated by co-expression of the pluripotency genes (Figure 1e–f”). Similar to the late neural fold stage data, we detected subpopulations of stem cells (light blue CNS; red NPB; light green NNE) that, based on the strongest expression of genes related to one of the ectodermal domains, were already transitioning to an ectodermal fate while still co-expressing pluripotency markers as well as markers of the neighboring domains. Spatial back-mapping showed that the transitioning stem cell domains overlapped with each other with intermingling cells appearing between domains, whereas the committed groups were clearly separated (Figure 1f–f”). Furthermore, this earlier stage contained a large stem cell population (orange), which based on the co-expression of all respective ectodermal domain markers consisted of cells that were uncommitted to a specific fate. Spatial back-mapping of these undecided stem cells into their respective original tissue locations showed that they spanned all three ectodermal domains (Figure 1f and Figure S1e). Finally, at the earliest stage examined immediately after gastrulation (HH5), we found that the majority of ectodermal cells clustered into the undecided stem cell group (orange) that spanned the entire ectoderm. Although some cells were already committed to the neural lineage and situated in expected medial position, or presented a transitioning transcriptional profile in line with findings on early spatial patterning^13,14^, most of the cells co-expressed neural and non-neural markers and had a pluripotent-like undecided stem cell profile despite being spatially located either medially or laterally (Figure S1f-f’). In sum, these results suggest that the expression of pluripotency genes is not abruptly lost after gastrulation, but rather is maintained in the ectoderm until late neurulation stages. Importantly, the spatial distribution of the subgroups was consistent in all analyzed parallel embryos as shown by mapping back the pseudo-colored cells into the respective original images (Figure S1c-f’). Our findings thereby suggest that the loss of stemness proceeds in concordance with a much slower sepration of the ectodermal domains than previously thought, consistent with recent scRNAseq and imaging reports of a gradual ectodermal patterning process that is completed only near the end of neurulation stage^15–17^.

### Pluripotency is not lost at the end of gastrulation but is maintained throughout the ectoderm

**Figure 2 |:**
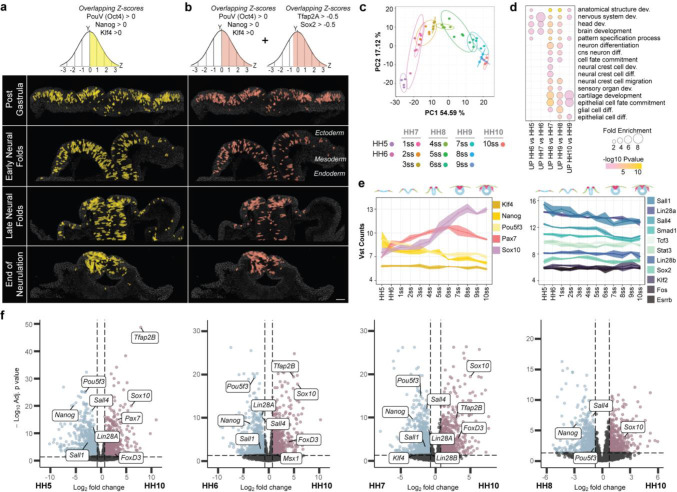
Pluripotency genes are maintained from gastrulation to the end of neurulation. **a**, Cells that have z-scores for all 3 pluripotency genes (*Nanog, PouV/Oct4 and Klf4*) above the mean are selected and pseudo-colored using scMST. Their visualization (yellow) highlights maintenance of stemness from gastrula stage in the entire ectoderm until end of neurulation when pluripotency is detected only in the dorsal neural tube. **b**, Similarly, pan-ectodermal stem cells co-express pluripotency genes together with the respective non-neural ectoderm and neural markers *AP2A and Sox2* (pink). **c**, Principal component analysis shows separation of bulk RNAseq data collected from NC domains according to developmental stage. **d**, Differentially expressed gene ontology term representations from bulk RNAseq data reflect NC development. **e**, VST (variance stabilizing transformation) normalized counts plotted on linear graphs across all stages, indicating a continuation of pluripotency gene expression with no gaps from gastrula to end of neurulation. Scale bar, 30 μm. **f**, Volcano plots of the Wald test show significantly upregulated (red) and downregulated (blue) genes at HH10 compared to earlier NC stages (p-adj <0.05, LFC 0.75), and stem cell genes from e are labeled along with NC markers.

As with any hierarchical clustering algorithm, the heatmap subgroups allow a certain level of heterogeneity in individual gene expression levels within a cluster, revealing important information on the expression of the stem cell genes (Figure 1e and Figure S1c-f). First, the pluripotency factors did not exclusively cluster together at any developmental time point suggesting their expression patterns are not identical and that each gene may play additional individual roles during early development that does not require co-expression with other pluripotency markers, as previously known from several other contexts^18,19^. Second, consistent with a known role in neural induction^20^, ectodermal Sox2 expression clustered with neural progenitor markers and therefore was considered a neural stem cell rather than a pluripotency marker. Thus, we focused on *Nanog*, *PouV /Oct4,* and *Klf4* co-expression to monitor pluripotency due to their known roles in epiblast and embryonic stem cell pluripotency^8,9^. We reasoned that if stemness were to be maintained from blastula stage onwards, continuous co-expression of the pluripotency genes would be readily detected. We therefore selected cells with scMST z-scores above the mean for all three pluripotency genes simultaneously. The results show that at the end of gastrulation (HH5), the ectoderm is comprised of numerous cells expressing a pluripotent signature and that these cells are maintained broadly throughout the entire ectoderm until the neural fold stage. Spatial restriction of these stem cells to the dorsal neural tube was only detected at the end of neurulation (Figure 2a and Figure. S3a), indicating that stemness is not restricted to the NC domain during the period between gastrulation and neural fold closure.

The expansive expression of stemness genes throughout the ectoderm led us to speculate that the purpose of these presumably plastic “pan-ectodermal stem cells” might facilitate a gradual patterning of the ectoderm by maintaining competence to form all ectodermal domains. To test this, we next utilized a similar approach to that described above, ie. scMST z-scores were used to select pan-ectodermal cells that co-expressed the three pluripotency genes together with the NNE marker *TFAP2A* and the future CNS marker *Sox2*, the two transcription factors that were also recently shown to co-bind during neural crest development^21^. The results show that ~70% of the pluripotent cells (Figure 2a) also displayed a pan-ectodermal gene expression profile and were found in all the ectodermal domains until being restricted to the dorsal neural tube at the end of neurulation (Figure 2b and Figure S3b-c). It is worth noting that while the majority of the pan-ectodermal stem cells reside in the NC domain at the end of neurulation, some cells spatially overlapped with the neural stem cells thereby highlighting their plasticity (Figure 1d). Furthermore, maintenance of pluripotency genes appears to be unique to the ectodermal germ layer as they were not detected in the mesoderm or endoderm (Figure 2a).

These results prompted us to hypothesize that the pluripotency-like NC stemness is maintained in the ectoderm past the gastrulation stage by the continued co-expression of pluripotency genes. To test this, we used bulk-RNAseq to analyze dissected cranial NPB/NC regions from embryos collected only õne hour apart from each other (to ensure detection of subtle transcriptional changes) from gastrula to end of neurulation stage (Figure 1a). As expected, the samples were readily distiunghished by developmental time (Fig. 2c), and accordingly, the gene ontology term representations, list of differentially expressed genes and overall change in gene expression reflected the early steps of NC development (Figure 2d and Figure S3d). Consistent with the scMST results, a linear graph shows continued expression of stem cell genes including the three pluripotency factors *Nanog*, *PouV/Oct4* and *Klf4* chosen for scMST. As no gaps were detected between the twelve stages overarching the neurulation process (stage HH5-10ss), these data do not support the idea that pluripotency is reactivated. As a validation of the sample dissection, the expression of the early NPB marker Pax7 started to rise soon after gastrulation, and the expression of the NC specifier gene Sox10 became prominent at the late neural fold stage, as expected (Figure 2e left panel). Because pluripotency circuitries are well studied in embryonic stem cells, we next asked if other components of the pluripotency complex were expressed in the developing NC. For this, we plotted other genes obtained from an unbiased list of core pluripotency factors^10^ and the linear graphs showed continuous expression of eleven additional stem cell factors throughout the neurulation stages, thus further supporting our findings (Figure 2e, right panel). Additionally, volcano plots show that the pluripotency genes are differentially expressed throughout all stages of premigratory neural crest development as compared to the later, migratory NC stage (Figure 2f). In contrast, NC genes are differentially expressed between early (HH6) and late neural fold stage (4som/HH8), indicating they are not continuously expressed but rather activated at the late neural fold stage at the onset of NC specification and are thus not likely to maintain the expceptionally high NC stem cell capacity from gastrula stage as previously suggested^2^ (Figure S3e). The linear plots revealed a slight overall decrease in transcript numbers of the pluripotency genes during the neurulation process, which raised the question of whether the transcript levels are biologically relevant and translated into protein. Immunofluorescence confirmed that Nanog was readily detectable in the NC domain at the end of neurulation (Figure S3f). Together, scMST and bulk RNAseq analyses show that pluripotency is not lost in the ectoderm upon gastrulation but is maintained in the entire ectodermal germ layer as it is patterned into its functional domains. The high stemness, as evaluated by co-expression of core pluripotency genes, is eventually restricted to the dorsal neural tube (neural crest and neural stem cell domains) at the end of neurulation.

### scRNAseq supports scMST data on ectodermal pluripotency gene expression maintenance after gastrulation

**Figure 3 |:**
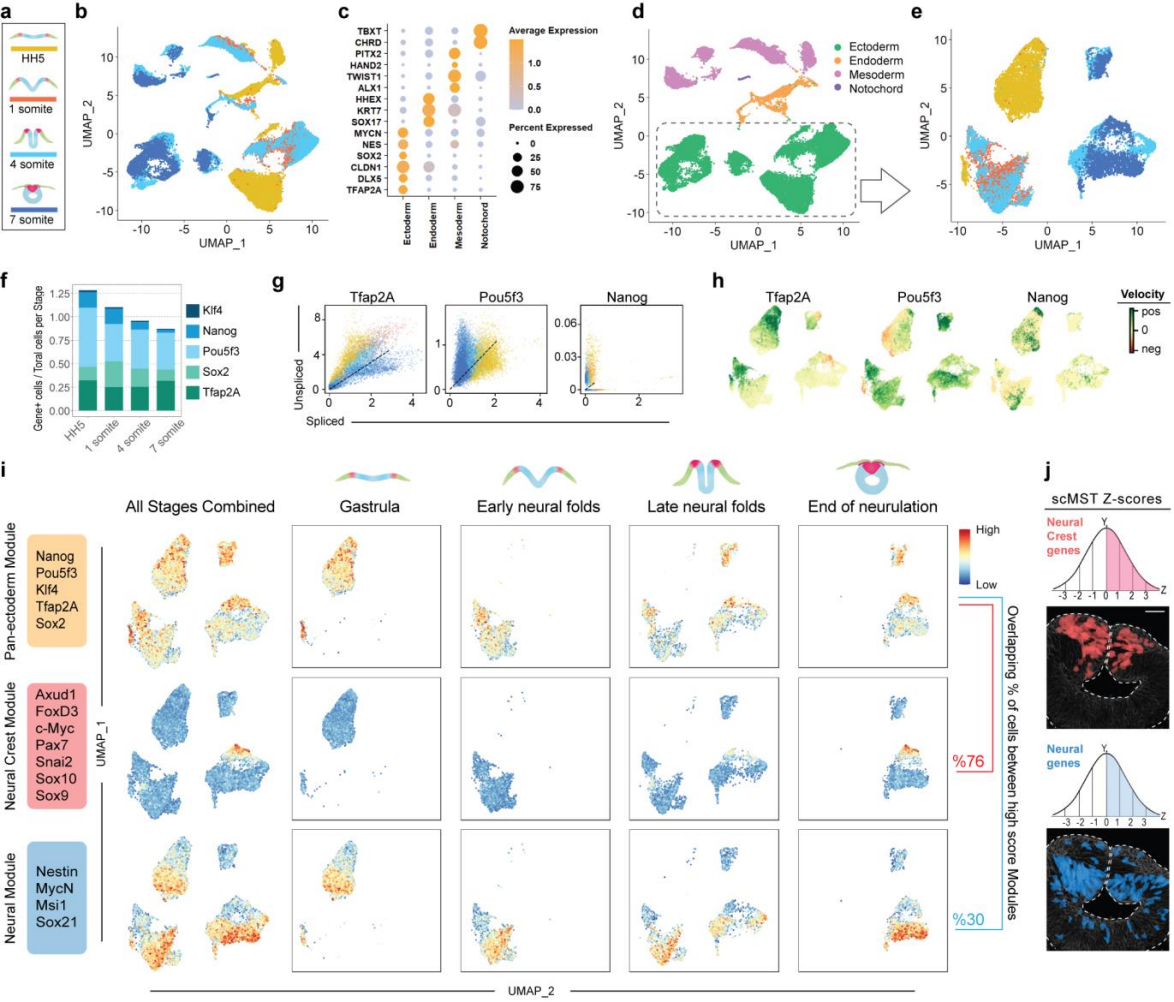
scRNaseq data substentiate maintenance of ectodermal pluripotency signature and pan-ectodermal stem cells. **a**, Color-coding according to developmental stage of scRNAseq samples in figures from b to g. **b**, UMAP of all data colored according to developmental stages. **c**, Based on enriched genes in each cluster shown in the dot plot, cells in the UMAP are separated according to germ layer identities. **d**, Re-colored UMAP representing each germ layer. **e**, Ectoderm cluster was subsetted for a detailed analysis. New UMAP is colored to show each developmental stage in the ectoderm group. **f**, Bar plots highlight continued expression of pluripotency genes in the cranial ectoderm from gastrula to end of neurulation. **g**, RNA velocity plots show a higher ratio of unspliced RNA for *Nanog* and *PouV/Oct4* throughout developmental stages indicating continuous active transcription, whereas *Tfap2A* transcripts at the end of neurulation are predominantly spliced. **h**, Positive velocity (green) in cells that show higher abundance of unspliced mRNA in UMAP. **i**, Modules reflecting Pan-ectodermal, NC and neural genes with their expression scores shown on the feature plots. 76% of the cells with high NC score, and 30% of the ones with high neural score also share high score for pan-ectodermal cells. **j**, Genes used in NC and neural modules are chosen from scMST data in order to visualize the cells that co-express the respective genes above the mean (z-score>0). Scale bar, 30 μm.

**Figure 4 |:**
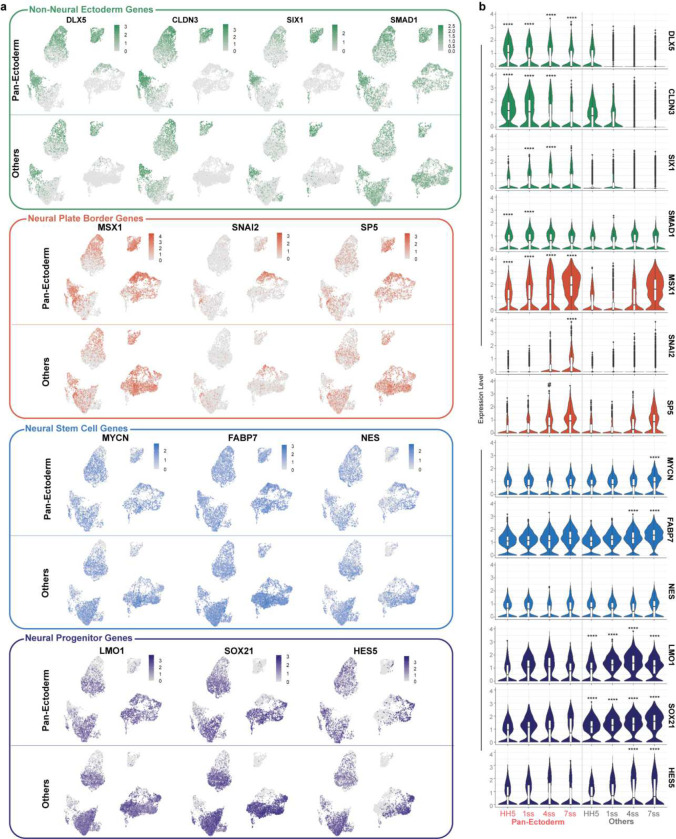
Transcriptional profiles of Pan-ectoderm vs “other” ectodermal cells **a**, Feature plots demonstrate the expression patterns of individual marker genes that represent different ectodermal domains from gastrula to closed neural tube stage in pan-ectodermal cells and the rest of the ectodermal cells (others). **b**, Violin plots demonstrate expression levels of individual genes. Black bar indicates median value. Non-neural ectoderm and neural plate border markers are predominantly expressed in the pan-ectodermal subset, whereas neural stem cell markers are readily expressed in both groups while more committed neural progenitor markers are predominantly expressed in the “others” population. Differentially expressed genes within a single developmental stage with p-adj values <0.0001 are marked with **** (logFC threshold= 0.2, min.pct= 0.25).

To further validate our results, we collected scRNAseq samples at the midbrain level from the equivalent developmental stages used for scMST and analyzed their transcriptomes by using the 10x genomics platform (Figure 1a, 3a). From two independent samples per stage, we captured 5–10K cells per sample for sequencing (Figure S4a-b). As expected, we identified cell clusters reflecting all three germ layers (Figure 3b–d, Figure S4c-d, Table S1). Consistent with the scMST results, feature plots revealed that pluripotency gene expression was largely restricted to the ectoderm (Figure S4c, e-f). For a higher resolution analysis of stemness genes during ectoderm development, we subclustered the ectodermal cells (Figure 3e). *Nanog*, *PouV/Oct4* and *Klf4* were continuously expressed in the ectodermal cells during neurulation (Figure 3f). To gain further understanding on the dynamics of the transcriptional process of the pluripotency factors, we took advantage of the RNA velocity algorithm that calculates the ratio between unspliced and spliced transcripts. A predominance of unspliced RNA is a sign of active transcription of the respective gene^22^. We reasoned that if stemness were maintained from the gastrula stage, we would expect to see high velocity for the pluripotency genes throughout the neurulation process. Indeed, pluripotency mRNAs were predominantly unspliced and positive velocity was thus found at all four developmental stages. In contrast, active transcription of the Tfap2A control gene showed reduced velocity at the end of neurulation as demonstarated by the predominance of spliced mRNAs (Figure 3g–h). Finally, we asked whether pan-ectodermal stem cells could be identified from the scRNAseq data set, similar to what we discovered with scMST. We used module scoring to visualize cells with a pan-ectodermal stem cell signature featured on the UMAPs of the ectodermal subset. As expected, we detected cells with a high pan-ectoderm score at all four developmental stages (Figure 3i). Next, we ascertained the locaton of the specified NC cells to learn whether the pan-ectodermal cells eventually reside in the NC domain at the end of neurulation as suggested by the scMST results (Figure 2b). Indeed, cells with a high score for the mature NC module, consisting of genes known to be expressed at the end of neurulation (premigratory) stage, showed a similar pattern to the pan-ectodermal cells. For spatial reference in the embryo, Figure 3j shows cells that co-express the same genes used in this NC module with above the mean scMST z-scores at the end of neurulation stage. Quantification showed that at the last stage assayed, 76% of the cells with a high score for the NC signature also had a high score for the pan-ectodermal stem cell signature (Figure 3i and Figure S4g). scMST revealed some pan-ectodermal stem cells also in the neural stem cell population in the dorsal neural tube (Figure 2a and 1d). To look for signs of this in the scRNAseq data, we created a module also for CNS neural cells (visualization of spatial expression domain of neural genes by using scMST is shown in Figure 3j). Indeed, 30% of the cells with a high neural module score also scored high for pan-ectoderm (Figure 3i and Figure S4g). Additionally, we investigated the differeces in the transcriptional profile of the pan-ectodermal stem cells as compared to the rest of the ectodermal cells (“Others”). As expected, pan-ectodermal cells readily expressed genes representing all three domains. While the NNE and NPB markers were differentially expressed in the pan-ectoderm population, neural stem cell markers are equally expressed in both populations. However, the “other” ectodermal cells show a predominantly neural profile with more committed neural progenitor markers (Figure 4a–b, Table S2), supporting the idea that NC forms from the pan-ectodermal population, and suggesting that the separation of the neural lineage from the pluripotent pan-ectoderml cells is initiated before the other domains diverge during the ectoderm patterning process, as also shown by scMST (Figure S1f). The scRNAseq data thus corroborate our scMST and bulk RNAseq results, which together indicate that stemness genes are maintained throughout the ectoderm during neurulation to maintain plasticity. We hypothesize that this is the underlying mechanism that enables the NC to maintain its exceptionally high stem cell capacity in the dorsal neural tube at the end of neurulation.

### Pluripotency genes regulate stem cell mechanisms to ensure NC development at all axial levels

**Figure 5 |:**
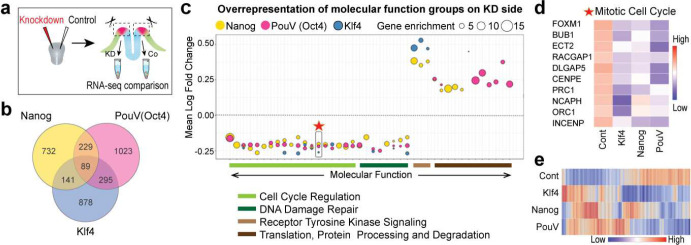
Knockdown of Nanog, Pouv/Oct4 and Klf4 affects stem cell functions in the ectoderm. **a**, Experimental design for knockdown by using two-sided injection of translation blocking morpholinos. **b**, Venn diagram depicting the number of genes that are differentially expressed due to individual knockdown of pluripotency genes. **c**, A weighed scatter plot highlights trends seen among the list of molecular functions in overrepresentation tests for each knockdown (KD) group as compared to pooled contralateral control sides. The gene enrichment (bubble size) depicts the number of differentially expressed genes found in a particular category and the number of times greater this pool size is compared to what is expected in a random list of genes. **d**, Heatmap showing an example of the genes included in the mitotic cell cycle function marked with a star in c. **e**, Heatmap highlighting the expression pattern of the 89 genes affected by knockdown of all three genes.

Our results suggest that co-expression of pluripotency factors Nanog, PouV/Oct4 and Klf4 in pan-ectodermal stem cells maintains stemness and allows plasticity during ectodermal patterning. However, the pluripotency genes may also have independent roles. To investigate this, we individually knocked down the three pluripotency genes using bilateral electroporation of a translation blocking morpholino on one side and a control morpholino on the contralateral side at gastrula stage (HH4) and analyzed the difference in gene expression in the NPB domains at neural fold stage (HH7/HH8−) by bulk RNA sequencing (Figure 5a). The samples (pool of 6–10 NPB domains per sample) segregated according to treatment (Figure S5a). Of all the 3387 differentially expressed genes, 89 were shared across each of the three knockdowns (Figure 5b, Table S3). The functional enrichment analysis revealed overrepresentation of 27 functional groups related to mitosis, cell cycle and DNA repair, in all of which the expression of genes relevant to the subgroup are downregulated in all three knockdowns, highlighted by an example heatmap of group 14 (marked with a star) consisting of mitotic cell cycle genes (Figure 5c–d). Of these functional groups, 30% (8/27) were downregulated following all three pluripotency gene knockdowns, and 78% (21/27) were downregulated following at least two of the knockdowns (Figure 5c). In contrast, several other cellular functions such as those related to translation, protein processing and degradation were primarily (67% ; 10/15) affected by loss of only one of the genes (Figure 5c and Figure S5b). Furthermore, individual molecular function over-representation plots also revealed general cell functions related to cell proliferation, growth, survival and stemness (Figure S5c-e). Overall, a heatmap depicting all the differentially expressed genes affected by knockdown of all three pluripotency genes supports the conclusion that while the outcomes caused by the individual knockdown of the three pluripotency genes are similar, unique gene-specific responses were also detected (Figure 5e). Additionally, regarding several cellular functions, different components of the same pathway are targeted by a different pluripotency gene, as shown in an example in Figure S5f. Combined, these results suggest that the pluripotency genes function to regulate cellular processes that together enhance stem cell capacity to ensure a proper ectodermal patterning process and neural crest formation.

**Figure 6 |:**
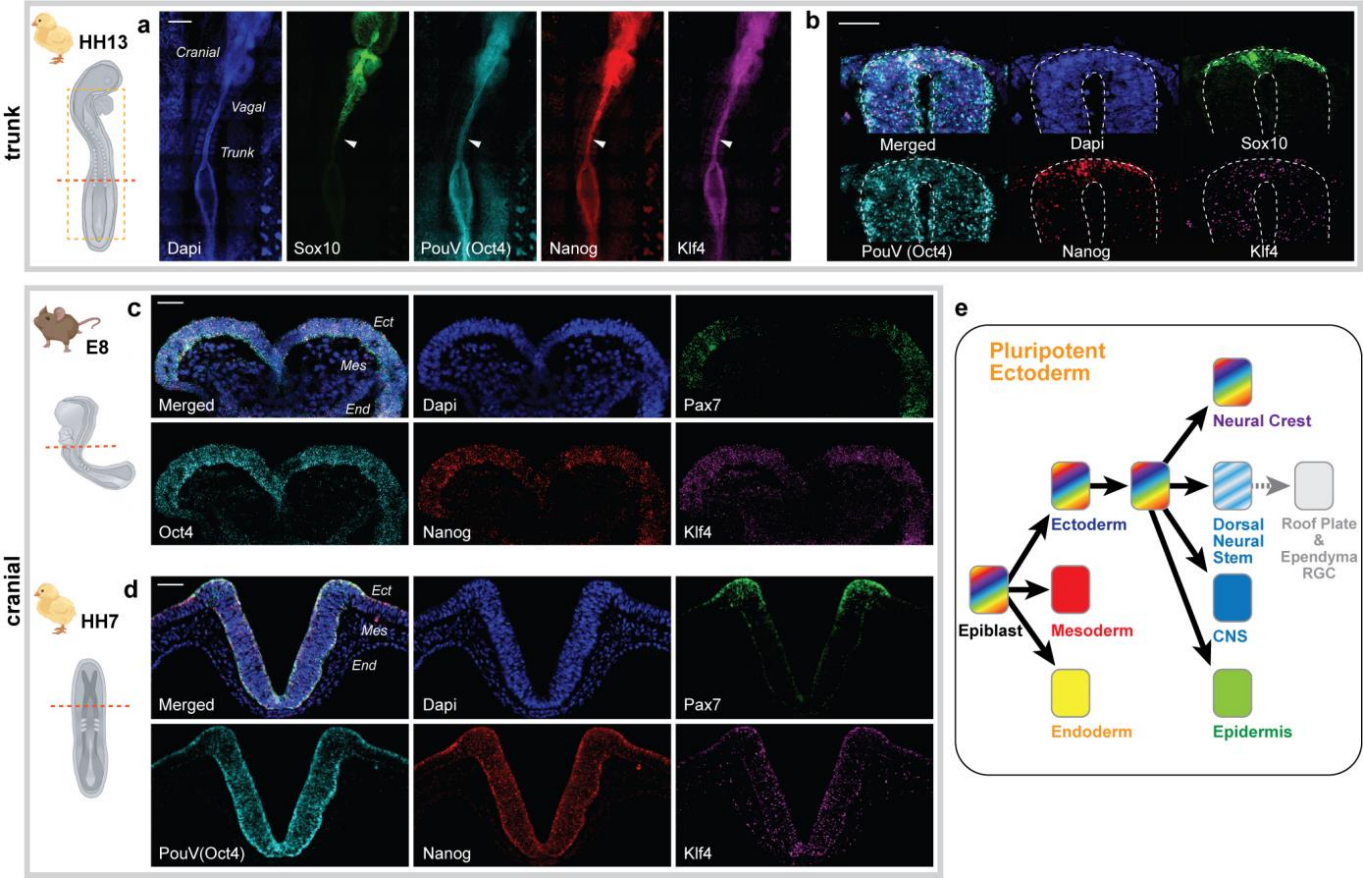
The pluripotency signature of the neural crest is detected at all axial levels, and broad ectodermal pluripotency gene expression is consistent across species. **a**, Whole mount fluorescent in situ hybridization shows expression of *Nanog (red), PouV/Oct4 (cyan)*, and *Klf4* (purple) in the trunk neural folds and plate of a HH13 chicken embryo. The arrow points to the end of neurulation level where NC is premigratory as indicated by the onset of *Sox10* (green) expression. Note that while co-expression of the pluripotency genes is restricted to the dorsal neural tube, *PouV/Oct4* and *Klf4* are expressed more broadly and may play additional roles during CNS development. Scale bar, 300 μm. **b**, Cross-section from the trunk level marked by arrow in A. Scale bar, 30 μm **c**, Cranial level cross-section from a mouse embryo at E8 shows expression of pluripotency genes in the entire ectoderm. Pax7 demonstrates the neural crest domain. Scale bar, 50 μm. **d**, Cranial cross-section of a HH7 chick embryo shows similar expression pattern of pluripotency genes as seen in mouse. Scale bar, 50 μm. **e**, A schematic depicting our hypothesis on ectodermal maintenance of pluripotency after gastrulation until the end of neurulation, highlighting the mechanism that enables NC formation.

These findings prompted us to hypothesize that co-expression of Nanog, PouV/Oct4 and Klf4 is a general requirement for NC development unrelated to the future lineage commitment of the NC cell. This is relevant since NC cells at different axial levels in the embryo are known to give rise to different cell types^1^. To test this, we performed multichannel fluorescent *in situ* hybridization (FISH) on older embryos (HH13, a stage during which the posterior neural folds are developmentally equivalent to the head region of a HH7-9 embryo) to address whether the pluripotency genes are expressed in the NC at all axial levels including the trunk, which does not give rise to mesodermal-like derivatives. Indeed, we detected continuous co-expression of pluripotency genes throughout anterior-to-posterior ectoderm (Figure 6a–b) suggesting that their expression does not dictate a specific NC fate. Additionally, cross-sections from the trunk level show that while individual *PouV* /*Oct4* and *Klf4* expression is detected in the entire developing neural tube, co-expression of all three pluripotency genes is restricted to the dorsal neural tube at the premigratory neural crest stage (Figure 6b), similar to our findings in the head region (Figure 1d).

Finally, studies on the mechanism of NC stemness formation have been performed in multiple model organisms, predominantly the mouse, frog, and chicken^2,3,5,7^. Since species-specific differences are known, we asked whether evidence for broad ectodermal maintenance of pluripotency exists in a mammalian model by performing FISH on equivalent developmental stages of mouse embryos. The results on cranial sections of (E8) mouse embryos show co-expression of *Nanog, PouV/Oct4* and *Klf4* throughout the entire ectoderm (Figure 6c) similar to the chick embryo (Figure 6d).

## DISCUSSION

We used single cell level gene expression analysis techniques to investigate both transcriptional and spatiotemporal changes reflecting cell fate commitment at the midbrain level during the ectoderm patterning process post-gastrulation. We find that pluripotency is not lost during gastrulation nor restricted to the NC domain. On the contrary, while mesoderm and endoderm lose pluripotency, we find cells with a pluripotent-like signature located throughout the ectodermal germ layer. The pluripotency signiture is co-expressed with both future CNS and epidermal domain markers, leading to their designation of “undecided pan-ectodermal stem cells”. We hypothesize that the continued co-expression of pluripotency genes is: 1) a requirement for maintaining cellular plasticity during the gradual patterning of the ectoderm, 2) the mechanism by which the NC domain acquires its exceptionally high stemness enabling it to form derivatives beyond “ectodermal capacity”, and 3) the mechanism by which the dorsal neural tube maintains plasticity as it transitions from NC to roof plate and ultimately the radial glia (neural stem cells) in the ependyma^23^. Our study using three independent high-resolution techniques proposes that stemness is retained from the blastula stage (Figure 6e) and thus resolves some of the controversy arising from previously published studies on NC stemness formation^2,3^. Our data demonstrating that NC specifier genes are first expressed at the end of neurulation, and that they are not continuously expressed in the ectoderm throughout neurulation, do not support a previous suggestion that the NC gene expression circuitry maintains stemness^2^. Furthermore, our finding that pluripotency genes are continuously expressed throught this period also does not support the idea that they are reactivated in the NC^3^. Instead, we propose that it is the continuous expression of pluripotency genes that is essential for the maintenance of stemness, which does align with the proposed principle idea of stemness maintenance from gastrula stage^2^, although our data reveals a different mechanism that is mediated by the pluripotency GRN and expands the stemess to the entire ectoderm rather than being restricted to the neural crest domain.

Importantly, we detect expression of pluripotency genes at all axial levels suggesting that they are broadly required for NC formation and ectoderm patterning. Furthermore, knockdown of the respective pluripotency genes affected overlapping gene regulatory circuitries involved in proliferation and stemness associated functions. These findings do not align with recent reports that link pluripotency-like features only to the anterior part of the embryo because of the unique ability of the NC to form the craniofacial skeleton in the vertebrate head ^3,5^. However, stem cell potential is not necessarily equivalent to the final selection of the cell types the stem cell ultimately produces. In other words, the full potential may not always be utilized. Although the NC does not contribute to ecto-mesenchymal derivatives in the trunk region of the commonly used animal models including chick, frog, mouse and zebrafish models ^24–29^, reports from turtle embryos show trunk NC-derived bone in the plastron^30^, and transplant studies of axolotl trunk NC have shown their contribution to the branchial arch cartilage^31^. These reports indicate that the high stem cell potential may be an ancestral feature of the NC, initially maintained at all axial levels but lost secondarily in different vertebrate embryo models, according to species-specific needs (see also review ^32^ for recent discussion). Furthermore, our findings do not rule out the possibility of additional, individual respective roles for the pluripotency genes later in NC development, such as supporting ecto-mesenchymal lineages at emigrating and migratory stage, as recently reported^3,5^. Thus, pluripotency defined by co-expression of core pluripotency genes is not to be confused with their individual expression, such as Oct4 detected both in the maturing neural ectoderm and mesoderm^18,19^.

Recent discoveries propose a role of pluripotency genes in participating the transition fom pluripotency to committed lineages by co-binding to lineage factors that redirect their binding to new enhancer sites, leading to regulatory interference from other factors inhibiting them to activate downstream targets, and eventually a gradual repression of the pluropotency gene transcription^21,33,34^. We show a comcomitant gradual loss of the pluripotency signature and increase in fate commitment during the ectodermal patterning process, suggesting possible involvement of similar epigenetic changes. On the other hand, neural crest cells do posses an exceptionally high pluripotent-like stem cell potential as judged by their ability to form derivatives beyond the ectodermal capacity. Our multiple lines of investigation combined argue for the original role of the pluripotency genes in enhancing stemness throughout the ectodermal patterning and neural crest specification process, allowing speculation of a dual role for the pluripotency factors in the ectoderm in both maintaining stemness and assisting gradual fate commitment. Future studies are also needed to investigate whether the neural crest cells truly are pluripotent or something in between pluripotency and multipotency (Pleistopotent^1^).

Finally, our unexpected finding of sustained, broadly distributed ectodermal co-expression of pluripotency genes long after gastrulation has ceased challenges the dogmatic viewpoints of gastrulation in which pluripotency is lost during the formation of the three germ layers. It is worth noting that the formation of ectoderm is very different from endoderm and mesoderm, which both ingress from the pluripotent epiblast (primitive ectoderm) and lose pluripotency in the migration process, while less is known about the detailed molecular signatures that induce the commitment of definitive ectoderm to form from the remaining epiblast layer, which continues to be maintained as an epithelial sheet^10^. Perhaps this morphological difference has enabled the entire ectoderm with the unique feature of maintaining pluripotency-like characteristics after gastrulation to ensure a faithful ectodermal patterning process, leading to formation of the NC in vertebrates.

## METHODS

### Chicken

Fertilized chicken eggs were obtained from the University of Connecticut (UCON) poultry farm (CT, USA) and Lassilan Tila (Tuusula, Finland), incubated at 37°C to reach the desired stage according to Hamburger and Hamilton (HH). See figure 1a for the exact stages used for each experiment.

### Mouse

Wild-type C57BL/6J mice were obtained from the Jackson Laboratory. For timed matings, noon on the day a copulation plug was detected was considered to be 0.5 days postcoitus. Embryos were collected in a sex-unbiased manner. All mice were maintained under specific pathogen-free conditions at the NIH animal care facility. Mice were cared for, and all experiments were approved by the Administrative Panel on Laboratory Care, and the Institutional Animal Care and Use Committee(IACUC) of NIH. Mice were maintained on a normal chow diet.

### Single Cell Multiplex Spatial Transcriptomics (scMST) Pipeline

The scMST pipeline was modified from our previously published technique Spatial Genomic Analysis ^6,7^, which was originally designed to analyze a single field of view at a single developmental time point. To govern analysis of multiple fields of views per sample and at multiple developmental stages, scripts that enable comparison of gene expression across all samples were added. Detailed instructions and codes are available in Github (https://github.com/KerosuoLab/Pajanoja_2023).

In brief, multiplexing is based on serial single molecule fluorescent in situ hybridization (smFISH) rounds on cryosections. For each gene of interest, up to 24 short DNA probes (minimum 13 probes) that target the coding sequence of the same mRNA were used. 3’ end of each probe was linked with a HCR initiator sequence (B1, B2, B3, B4 or B5; ^35^) via a 4 nt long linker sequence resulting in a 60 nt-long probe. Expression of five genes was captured on each round using a spinning disc microscope with six channels. At the end of each round, signal was removed by using DNase I, followed by another round of hybridization by using probes to five new genes applied on the same sample. All hybridization and imaging steps were done in hybridization chambers (Grace bio labs, 50ul) that were mounted on the coverslips. As the last step of the imaging rounds, cell borders were captured by using immunolabelling, which is critical for the machine learning algorithm-based 3D cell segmentation and quantitative dot counting of individual RNA transcripts within each single cell to work.

For the scMST analysis, each cell with quantitative expression data of the thirty genes was pooled into a heatmap that clusters them into unbiased subpopulations according the transcriptional profile. The spatial location of the cells within the subpopulations of interest was then pseudo-colored and visualized on the original tissue section images allowing maintenance of the original spatial context of the sample. The following specific changes were made to the original pipeline: First, in order to minimize uneven illumination issues, which occur due to artefacts in the imaging process, images were pre-processed using using the two-step filtering macro command ^36^. In the first step, the macro utilizes Fiji BaSiC plugin, a retrospective image correction method used to correct uneven image illumination ^37^. The plugin corrected flat field in each channel by performing low-rank and sparse decompositions. The second step uses Fiji 3D median filter with 3×3×3 pixel kernel to reduce noise and enhance spot detection. Second, as means to achieve unbiased signal selection, the previous signal processing step that excludes unspecific bound probes (displayed as low intensity) was replaced with K-means clustering, an unsupervised machine learning algorithm that clusters dots based on their signal intensity, providing a more robust approach (Figure S1A). Third, the Matlab code was further optimized for samples with multiple fields of views. Samples were z-scored across all three fields of views, therefore providing a more accurate and consisting level of “high” or “low” expression of a given gene within each cross section. Stage by stage analyses (stage specific heatmaps in Figure 1d–f and Figure S1) were done in this manner. Additionally, z-scores across all developmental stages were calculated by pooling all samples, and then z-scores were calculated through genes to gain understanding of expression levels of each gene between different developmental time points Figure S2).

Selection and pseudo-coloring of cells that have a z-score > 0 (higher than the mean) was used to determine cells with overlapping pluripotency gene expression (PouV / Oct4, Nanog and Klf4 in Figure 2a) as well as highlighting pan-ectodermal stem cells (PouV /Oct4, Nanog, Klf4, TfAp2A and Sox2 in Figure 2b). Similar method was used to highlight neural crest (Foxd3,c-Myc, Pax7, Snai2, Sox10, Sox9), and neural cells (Nestin, MycN, Msi1) to complement module scoring in figure 3j. The data set was filtered for the respective genes with a z-score > 0 and the cell IDs that show overlapping filter results for all respective genes of interest were intersected.

### Coverslip coating

The following changes were made to the sample preparation as compared to our previously published protocol ^6,7^. Glass surface preparation and silane coating of the slides was done as described ^38^. Briefly, slides were incubated in 50% (v/v) nitric acid for 25 min, followed by 200nM NaOH for 15 min to make glass surface more conductive for silane binding. Silanization of the slides was performed with 1% (w/v) Triethoxysilylbutraldehyde (TESBA) made in ethyl alcohol for 5 min at room temperature. The slides were rinsed with 100% ethanol twice and once with distilled water, and heat-dried in an oven at 64 °C for 4h or overnight. Next, the slides were treated with (0.1 mg/ml) Poly-l-lysine (Sigma) in PBS for 20 min at room temperature, followed by three rinses with PBS. The coverslips were then air-dried and kept at 4 °C in an airtight storage for no longer than 4 weeks.

### Chick embryo sample collection

For scMST, samples were collected on Whatman filter papers, fixed in 4% PFA overnight, and washed 3 times with PBS-0.2% triton, dehydrated in ethanol and stored in −80°C. The embryos we incubated through a sucrose gradient (5% 30 min, 15% 4h at 4°C and embedded into OCT as previously described ^6,7^ and 20 μm thick transverse cryosections were cut from the midbrain level onto silane coated coverslips.

For bulk RNAseq experiments, embryos were collected on Whatman filter papers in PBS and midbrain level neural plate border and specified neural crest cells, respectively, from left and right sides were manually microdissected using tungsten needles, immediately placed into RNA Lysis Buffer (RNAqueous^™^-4PCR Total RNA Isolation Kit, Thermofisher, Waltham, MA, USA) and stored at −80 °C. Replicates consisted of pooled tissue from at least five to seven embryos. Four replicates were collected per stage. For HH5 and HH6 replicates, rectangular patches lateral to the primitive streak were collected according to midbrain neural crest fate mapping as described ^13^. For premigratory stages beyond HH6, neural plate borders or neural fold apices corresponding to the midbrain neural crest domain were excised. Beyond 7ss, dorsal neural tubes containing neural crest from the midline to migratory front were collected.

The scRNAseq samples were collected by dissecting midbrain slices (covering all germ layers) from the desired stage by using micro scissors, which were pooled from three to four embryos per sample and dissociated with a multi tissue dissociation kit (kit 3,130-110-204, Miltenyi Biotec,) at 37°C for 20 min with intermittent pipetting to achieve a single cell suspension.

### Immunostaining of cell membranes:

A cocktail of E-cahderin (BD Transduction 610181), B-catenin (Abcam ab6391) and N-cadherin (MNCD2, Developmental Studies Hybridoma Bank, AB_528119) primary antibodies were used to label the cell membranes. Secondary antibodies were AlexaFluor647 for E-cahderin and AlexaFluor488 for B-catenin and N-cadherin.

### Imaging:

Imaging was performed using an Andor Dragonfly 200 spinning disk confocal system coupled to a Zeiss AxioObserver (Zeiss, Thornwood, NY). Coverslips were placed onto sealed coverglass rectangular chamber (ASI Imaging, I-3078-2450) in order to minimize errors for aligning images in each imaging round. A 63X apochromat water immersion objective (NA 1.27) was used for imaging cross sections. An Andor integrated laser engine provided the excitation light using 405 nm (100 mW), 488 nm (150 mW), 561 nm (100 mW), 594 nm (100 mW), 640 nm (140 mW), and 730 nm (30 mW) laser lines, and using suitable emission wavelengths for each fluorophore (DAPI, AF 488, Cy3B, AF 594, Alexa 647, and Cy7), respectively. A Photometrics Prime 95B CMOS (Photometrics, AZ) camera was used in 12-bit mode with 3X gain (13X 13 μm pixels). Exposure times and relative laser intensity was varied based on sample/fluorophore brightness (~ 500 ms and ~40% for each fluorophore). A Z piezo stage (ASI Imaging, Eugene, OR) allowed rapid imaging in Z. Images were collected every 0.5 μm over a 25 μm distance. All components were controlled by Micro-manager version 1.4.22 using the Andor driver and was programed by ADD. DAPI staining was imaged during the first round in addition to the five sets of probes (405 nm). For the final round of immunostaining, Alexa647 (640 nm) for E-cadherin with 500 ms with 20% laser power, and Alexa488 (473 nm) for B-catenin and N cadherin cocktail was used with 400 ms 20% laser power. Each cross section was captured using 3 fields of frames (boxes with dashed lines in Fig. 1a) with the help of the automated position software built in the microscope. All images were aligned in our custom Matlab script by overlapping images from each hybridization round and choosing the coordinates for the ideal alignment.

### Morpholino knockdown and RNA extraction for bulk RNAseq

Translation blocking morpholinos were designed to target the 5’ UTR in close proximity of the ATG for the respective genes: Nanog (CATGGTCGGGACGACACCTCCAG), PouV (CCGAGAGCTGCCTCCATGCTA), and Klf4 (CCGTCGTCCCGCCGAGGAGAGT). A control morpholino was designed to account for non-specific effects from electroporation (CCTCTTACCTCAGTTACAATTTATA). Morpholinos were conjugated on the 3’ end with carboxyfluorescein. Morpholinos were diluted to a 1.0 mM concentration and electroporated together with an empty pCIG vector as carrier DNA (1 μg/μL). Injections were two-sided, targeting the ectoderm in HH stage 4 chicken embryos, with control morpholino on the contralateral side. Chicken embryos were collected on Whatman filter papers and electroporated using 5.3V and 5 pulses (50mA/100mA), as previously described ^39^. Embryos were then incubated on individual petri dishes in thin albumin until they reached HH7 (1–3 somites). Embryos were then dissected to isolate the dorsal neural folds, which were immediately placed into lysis buffer. RNA was extracted from the tissue using the RNAqueous-4PCR Total RNA Isolation Kit (AM1914) according to kit directions. Extracted RNA was then ethanol precipitated and resuspended in nuclease free water before storing at −80°C.

### RNA sequencing analysis

#### Bulk RNAseq for 12 developmental stages

Bulk RNA-seq was performed by the NIDCD/NIDCR Genomics and Computational Biology Core (GCBC). Libraries were created using the Nextera XT method. Samples collected from 12 different developmental stages were run on an Illumina NextSeq 2000 configured for 55 PE reads. The counts were generated by mapping via STAR aligner (v2.5.2a). Reads are 55×55bp in length, and samples contain between 43.7and 87.5 million uniquely mapped reads to gene regions. Counts were filtered to remove all genes which did not have more than 5 reads in at least 1 of the samples. Differential expression analysis was carried out using DESeq2 ^40^. Samples were pooled as shown in Figure S3d, and for Figure 2f, differential gene expression analysis using the Wald test was performed comparing HH10 to earlier stages separately , and for comparison of HH8 to HH6 in Figure S3e (P-adj value < 0.05, LFC threshold 0.75). The DAVID ^41,42^ resource tool was used to find Gene Onthology (GO) terms using up-regulated genes (LFC > 0.75). Fold enrichments were obtained using statistical overrepresentation test, and p-values were calculated using Fisher’s Exact test and adjusted using the Bejamini-Hochberg false discovery rate (FDR) method for multiple test correction. For linear plots in Figure 2e, variance stabilizing transformation (VST) was calculated by using the earliest developmental stage in each comparison as a reference level using “relevel” function.

#### Bulk RNAseq for morpholino knockdown of pluripotency genes

Gene counts were produced by the NIDCD/NIDCR Genomics and Computational Biology Core using the STAR aligner in standard mode. The reads were mapped against a current ENCODE chicken release and the mapping parameters were derived from the GENCODE project. Counts were filtered to remove all genes that did not have more than 5 reads in at least 1 sample. Then counts were normalized by library size and analyzed with principal component analysis (PCA). A PCA1/PCA2 graph revealed an apparent batch effect preventing meaningful interpretation of the read counts, which was removed by using the RUVseq (remove unwanted variation) ^43^ function as follows: Remove Unwanted Variation with replicate Samples (RUVs) was used to remove the batch effects by using factor analysis on the control samples. Additionally, one thousand in-silico control genes, identified as those with the lowest coefficient of variance among all samples, were passed to the function. Transformation of the data using RUVs revealed a PCA1/2 plot showing clear gene expression signatures for each KD group and control group. After normalization, differential gene expression (DGE) analysis was performed using DeSeq2, where each KD group was compared to all control samples. This revealed many genes, which showed a statistically significant change (p-adj <0.05) in expression levels between the KD groups and control, most of which were unique to only one KD group. Up and downregulated genes for each morpholino list were used for the overrepresentation test via PANTHER (GO biological process) ^44^. Fold enrichments were obtained using statistical overrepresentation test, and p-values were calculated using Fisher’s Exact test and adjusted using the Bejamini-Hochberg false discovery rate (FDR) method for multiple test correction.

#### 10X Chromium scRNA-seq

Single cell sequencing was performed at FIMM Single-Cell Analytics unit supported by HiLIFE and Biocenter Finland. Single cell gene expression profiles were studied using 10x Genomics Chromium Single Cell 3’RNAseq platform. The Chromium Single Cell 3’RNAseq run and library preparations were done using the Chromium Next GEM Single Cell 3’ Gene Expression version 3.1 Dual Index chemistry. The Sample libraries were sequenced on Illumina NovaSeq 6000 system using read lengths: 28bp (Read 1), 10bp (i7 Index), 10bp (i5 Index) and 90bp (Read 2). Fastq files were generated by using the 10x Cell Ranger (v7.0.1) count pipeline of Cell Ranger as well as by performing alignment, filtering and UMI counting. Finally, Fastq files were mapped to chicken genome using a custom GRCg6a Gallus gallus genome annotation file that was constructed by extending selected 3’UTRs in order to account for incorrect annotations.

Data cleaning, normalization and scaling was performed as follows: downstream analysis was carried out using Seurat package (v3.0.1) ^45^ in R. SoupX ^46^ and DoubletFinder ^47^ packages were used to filter out ambient RNA and doublets, respectively. Cleaned samples were merged and named based on their original file names. Following the standard Seurat workflow, artifact cells were further excluded by removing any cells that expressed more than 4000 genes and high mitochondrial content (>0.4%). Gene expression matrices were normalized and scaled using the NormalizeData and ScaleData functions. During the scaling, Cell Cycle Difference scores, which was calculated by difference of S from G2/M-phase specific gene scores via ‘CellCycleScoring’ function, were regressed out. Then, principal component analysis linear dimension reduction was conducted using using the ‘ElbowPlot’ function (resolution 0.2, dims 1:15). Clustered cells were visualized via UMAP plot and manually annotated based on differentially expressed genes in each cluster (FindAllMarkers function, logfc.threshold= 0.2, min.pct= 0.25). The clusters that were identified as “Ectoderm” were extracted and re-clustered using the ‘subset’ function and given colors based on their individual stages. Bar plots were used to demonstrate expression levels of selected genes. Each bar represents cell counts that express the gene of interest individually, and the values were normalized by total cell counts in each developmental stage or germ layer, respectively. Module scores were calculated based on individual list of genes we have used, using the AddModuleScore function in Seurat. For in depth analysis, cells in the ectoderm UMAP were labeled based on module score as “Pan-ectoderm” (Pan-ecto score >0) vs “Others” (Pan-ecto score <0). Differentially expressed genes between Pan-ectoderm vs Others were calculated via stage by stage comparison (FindAllMarkers function, logfc.threshold= 0.2, min.pct= 0.25). Ratios of unspliced vs spliced RNA were calculated via scVelo dynamical modelling pipeline ^22^ in Python. For each sample, Loom files containing spliced and unspliced RNA expression matrices were created using the Velocyto.py command line tool. Ectoderm subset of HH5, 1 somite, 4 somite and 7 somite stages were selected for the analysis.

#### Antibody production

Custom Nanog and FoxD3 Monoclonal antibodies (Genscript Antibody Services, Piscataway, NJ) were commercially generated against full-length recombinant chicken proteins. Forty parental lines were received and tested by immunoblot and immunohistochemistry using chicken lysates and fixed embryos (FoxD3: HH9-10; Nanog: HH5-6 primordial germ cells). Select parental lines were clonally expanded and monoclonal hybridoma isotypes were confirmed (Pierce^™^ Rapid Antibody Isotyping Kit, Thermofisher).

#### Whole mount Fluorescent In Situ with Hybridization Chain Reaction

Chicken (HH6- HH15) and mouse embryos (E7.75 – E8.5) were fixed in 4% paraformaldehyde, washed with PBS/0.2% Tween (PBST), dehydrated in methanol, and stored at −20 °C. All experiments involving animals were approved by the NIDCR Institutional Animal Care and Use Committee. HCR split-initiator probe sets were purchased from Molecular Instruments (www.molecularinstruments.com) for chicken Nanog (NM_001146142.1), PouV (NM_001309372.1), Klf4 (XM_004949369.3), Pax7 (NM_205357.1), and Sox10 (NM_204792.1). In situ HCR v3.0 with split-initiator probes was done according to whole-mount chicken protocol ^48^ with hybridization overnight at 37°C and amplification overnight at room temperature. Prior to hybridization, later stage embryos (HH12-HH14) were permeabilized with 10 μg/mL proteinase K for 2.5 minutes. Following amplification, embryos were washed with 5xSSCT, stained with DAPI, and post-fixed with 4% PFA for 15 minutes at room temperature. Embryos were mounted using ProLong Gold Antifade Mountant and imaged using an Andor Dragonfly spinning disk confocal microscope. For sections, embryos were embedded in gelatin and sectioned at 20 μm.

## Data Availability

All data needed to evaluate the conclusions in this study are present in the main text and the supplementary materials. Any additional data reported in the main and supplementary figures are available upon request. MATLAB scripts for scMST pipeline and R scripts for all scRNAseq analysis have been deposited into GitHub (https://github.com/KerosuoLab/Pajanoja_2023). Bulk RNAseq and scRNAseq datasets have been deposited to in the NCBI GEO database under accession GSE221125 and GSE221188 respectively.
